# Estimation of the molecular vibration of gases using electron microscopy

**DOI:** 10.1038/s41598-017-16423-0

**Published:** 2017-12-12

**Authors:** Hirotaka Katsukura, Tomohiro Miyata, Manabu Shirai, Hiroaki Matsumoto, Teruyasu Mizoguchi

**Affiliations:** 1 Institute of Industrial Science, The University of Tokyo, 4-6-1 Komaba, Meguro, Tokyo, 153-8505 Japan; 2 Hitachi High-Technologies Corporation, 24-14, Nishi-shimbashi 1-chome, Minato-ku, Tokyo, 105-8717 Japan

## Abstract

Reactions in gaseous phases and at gas/solid interfaces are widely used in industry. Understanding of the reaction mechanism, namely where, when, and how these gaseous reactions proceed, is crucial for the development of further efficient reaction systems. To achieve such an understanding, it is indispensable to grasp the dynamic behavior of the gaseous molecules at the active site of the chemical reaction. However, estimation of the dynamic behavior of gaseous molecules in specific nanometer-scale regions is always accompanied by great difficulties. Here, we propose a method for the identification of the dynamic behavior of gaseous molecules using an electron spectroscopy observed with a transmission electron microscope in combination with theoretical calculations. We found that our method can successfully identify the dynamic behavior of some gaseous molecules, such as O_2_ and CH_4_, and the sensitivity of the method is affected by the rigidity of the molecule. The method has potential to measure the local temperature of gaseous molecules as well. The knowledge obtained from this technique is fundamental for further high resolution studies of gaseous reactions using electron microscopy.

## Introduction

Chemical reactions that occur in gaseous phases and at gas/solid interfaces, such as those on catalysts and in fuel cells, hold a prominent position in modern industry^1–5^. The efficiency of gas-based chemical reactions is influenced by the gaseous temperature at the active site of the reaction; this effect is attributed to the dynamic behavior of the gaseous and adsorbed molecules, namely their rotational, vibrational, and translational kinetic energies. Measurement of the dynamic behavior of gaseous molecules with high spatial resolution is thus likely to facilitate the further development of efficient gas-based reaction systems.

Among the analytical methods, environmental transmission electron microscopy (ETEM) enables the *in-situ* observation of chemical reactions of gaseous phases at atomic resolution, and has been used in many studies of catalytic reactions, nanoparticle growth processes, and partial pressure measurement under several gas conditions^6–10^. In addition, electron energy-loss near-edge structure (ELNES) analysis with TEM provides chemical and bonding information^11–15^. Thus, the measurement of ELNES using ETEM has great potential for the identification of the dynamic behavior of gaseous molecules on the nanometer scale. However, identification of the dynamic behavior of gaseous molecules using ELNES has not yet been achieved owing to difficulties in interpreting the spectra. Especially, the relationships between the spectral features and the dynamic behavior of gaseous molecules have not been revealed.

In this study, we have developed a method for estimating the dynamic behavior of the gaseous molecules combining the ETEM-ELNES and the theoretical calculation. In the ETEM-ELNES experiment, vibration of the gaseous molecules was stimulated by heating the gases, and the changes in the molecular vibrations were identified using ELNES. The relationships between the spectrum and the dynamic behavior of the gaseous molecules were revealed by combining a first-principles calculation and a molecular dynamics (MD) simulation. The present study provides fundamental information for further high resolution studies of gaseous reactions using electron microscopy.

## Results and Discussion

**Figure 1:**
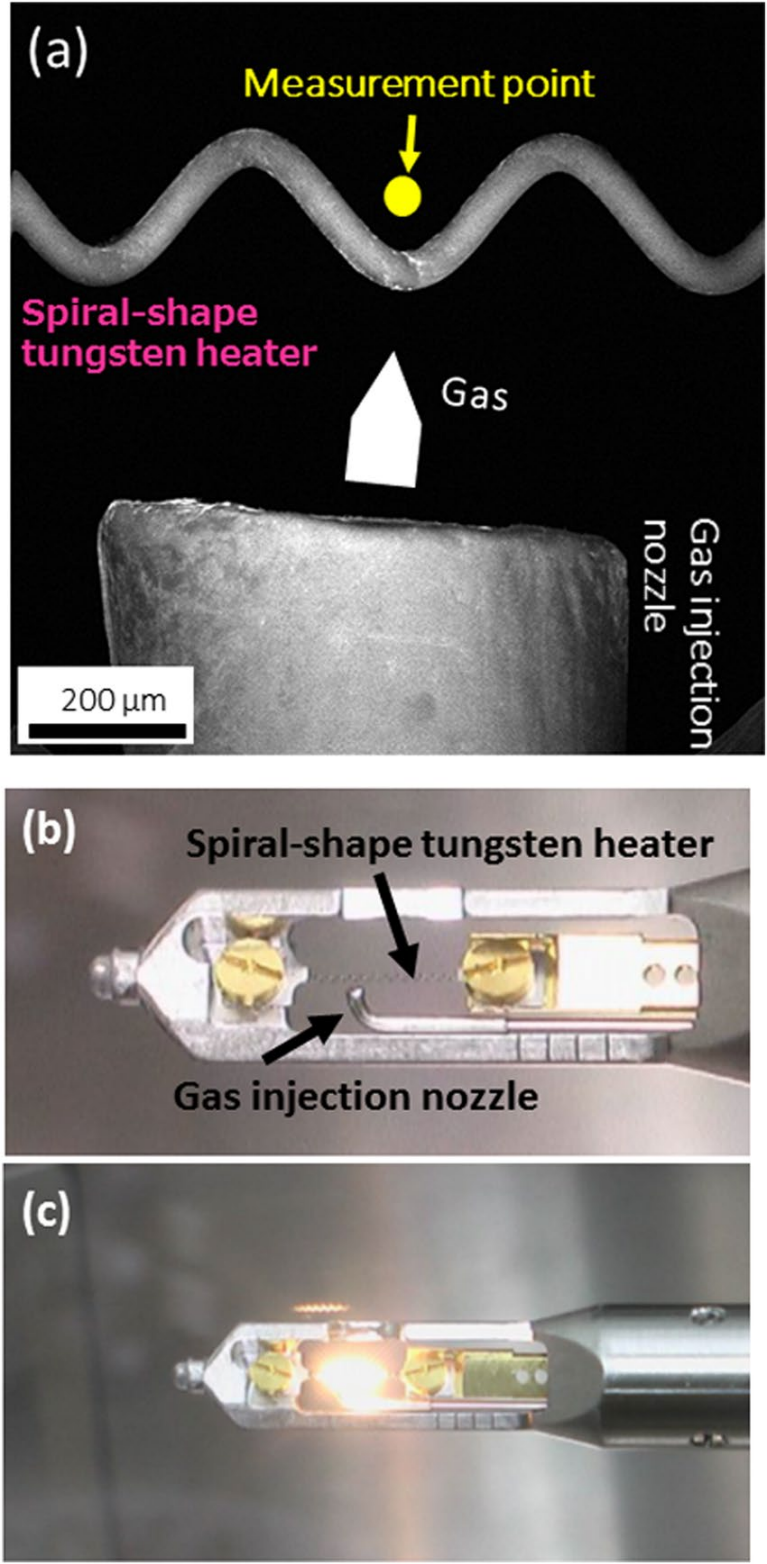
(**a**) Secondary electron STEM image near the gas nozzle and spiral-shape tungsten heater. Photographs of the gas-injection heating holder at (**b**) room temperature and (**c**) high temperature.

**Figure 2:**
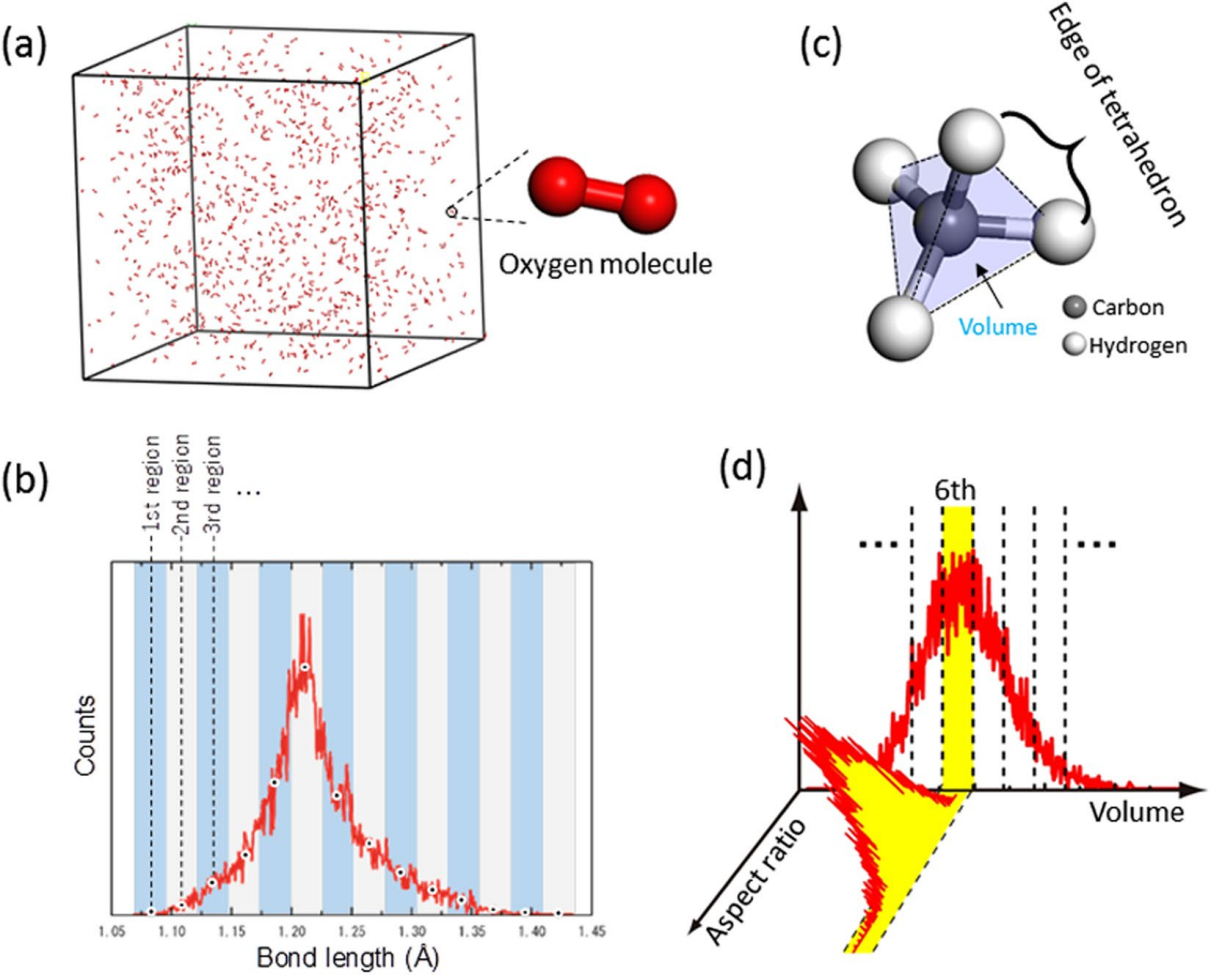
(**a**) Gas model for O_2_ at high temperature constructed by a molecular dynamic (MD) simulation. (**b**) Histogram of the O=O bond lengths in the O_2_ gas model in (**a**). (**c**) Stable molecular structure of CH_4_. (**d**) Schematic diagram of the histograms of the molecular volume and the aspect ratio for CH_4_.

ELNES from gaseous molecules were observed using a ETEM equipped with a gas differential pumping system and a gas-injection heating specimen holder^16^. Figure 1(a) shows the secondary electron-STEM image around the spiral-shape tungsten heater and gas nozzle. The measurement point is behind the heater as seen from the gas nozzle. To obtain the vibrational information from the experimental spectrum, we performed theoretical analysis using MD^17^ and the first principles simulations^18,19^. The method for calculating the ELNES of the gaseous molecules are schematically shown in Fig. 2 and described in the Methodology part in details. Hereafter, for simplicity, room temperature (298 K) and high temperature (1,273 K) are represented as RT and HT, respectively.

**Figure 3:**
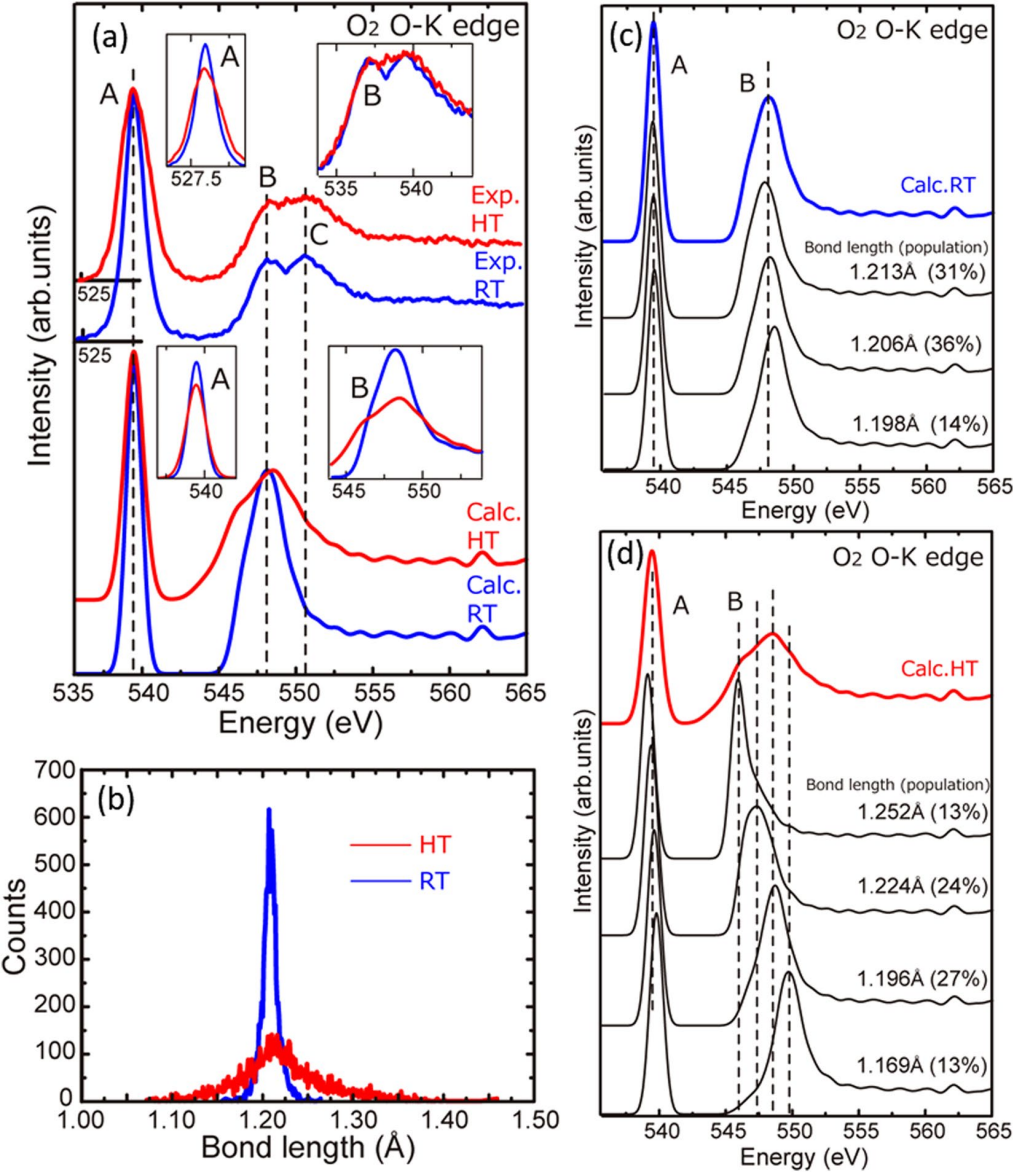
(**a**) Experimental and calculated O–K edges of O_2_ gas at room temperature (RT: blue line) and high temperature (HT: red line). (**b**) Histogram of the O=O bond lengths at RT and HT. (**c**) Calculated O–K edge spectra for representative O_2_ molecules with O=O bond lengths of 1.198, 1.206, and 1.213 Å. These molecules were taken from the gas model at RT. The numbers in parentheses are the populations of the respective bond lengths. (**d**) Calculated O–K edge spectra for representative O_2_ molecules with O=O bond lengths of 1.169, 1.196, 1.224, and 1.252 Å, which were taken from the gas model at HT.

Figure 3(a) shows the experimental and calculated O–K edges of O_2_ gas at RT (blue) and HT (red). In the insets, the intensities of peaks A and B and those of the higher energy side of peak C are aligned to allow a comparison of the intensity differences. We note that our calculation could not reproduce peak C because it originates from a Rydberg state related to the decay process of the excited electron^20^. Irrespective of the temperature, all spectra are composed of a strong peak A at the threshold, followed by smaller peaks B and C. However, detailed inspection revealed small but clear differences between the RT and HT spectra; the peaks become broader and less intense at HT, as shown in the insets. In addition to the intensity difference, the features of peaks B and C are obscured as a result of the broadened features. Our theoretical calculation reproduced these experimental spectral changes. Here, it should be mentioned that the spectral difference in the calculation is relatively larger than that in the experiment. This point will be discussed later.

To understand the cause of the spectral changes, the molecular structures in the gas models were investigated. The histograms of the O=O bond length for the RT and HT models are shown in Fig. 3(b), and show very different distributions depending on the temperature. At HT, the bond lengths show a larger dispersion than at RT, which indicates that molecular vibration is more intense at HT than at RT. Individual spectra for different bond lengths at RT and HT are shown in Fig. 3(c) and (d). For RT, O–K edges for O_2_ molecules with three different bond lengths (1.213, 1.206, and 1.196 Å), which represent the main portions of the histogram (31%, 36%, and 14%, respectively), are shown. The three molecules exhibit very similar profiles owing to their similar bond lengths. In contrast, at HT, the spectral features of molecules with bond lengths of 1.252, 1.224, 1.196, and 1.169 Å are clearly different (Fig. 3(d)), with the peaks shifting to higher energy as the O=O bond length decreases. In addition, the size of the shift is larger for peak B than for peak A. The larger shift of peak B compared with that of peak A occurs because peak A and peak B originate from π- and σ-type orbitals, respectively. The large shifts of peak B induced by intense molecular vibration thus result in the broader peaks at HT.

**Figure 4:**
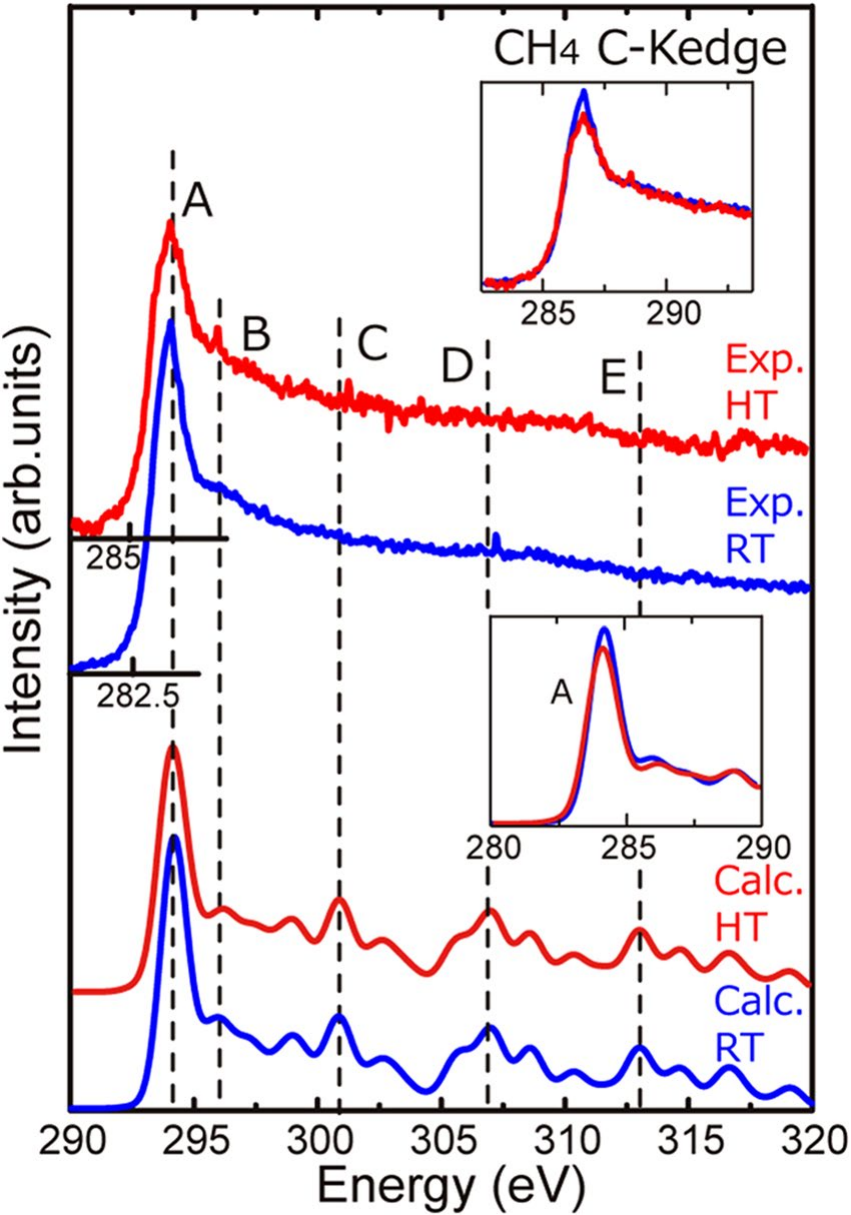
Experimental and calculated C–K edges of CH_4_ gas at room temperature (RT: blue line) and high temperature (HT: red line).

We confirmed that the identification of the dynamic behavior of CH_4_ gas is also possible using the present method. Figure 4 shows the experimental and calculated C–K edges at both RT and HT. This spectral feature is different from the O–K edge of O_2_ gas; the spectrum has an intense peak A, which is followed by broad profiles of peaks B to E. The theoretical calculations reproduced the characteristic features of the experimental spectrum. Similar to the O_2_ case, a small but clear temperature effect was observed, namely, peak A became broader and less intense at HT, as shown in the insets of the figure. Here, the spectral difference in the experiment is well reproduced by the calculation. This is different tendency from the O_2_ case, in which the spectral difference in the calculation is relatively larger than that in the experiment (Fig. 3(a)). This point will be discussed later.

**Figure 5:**
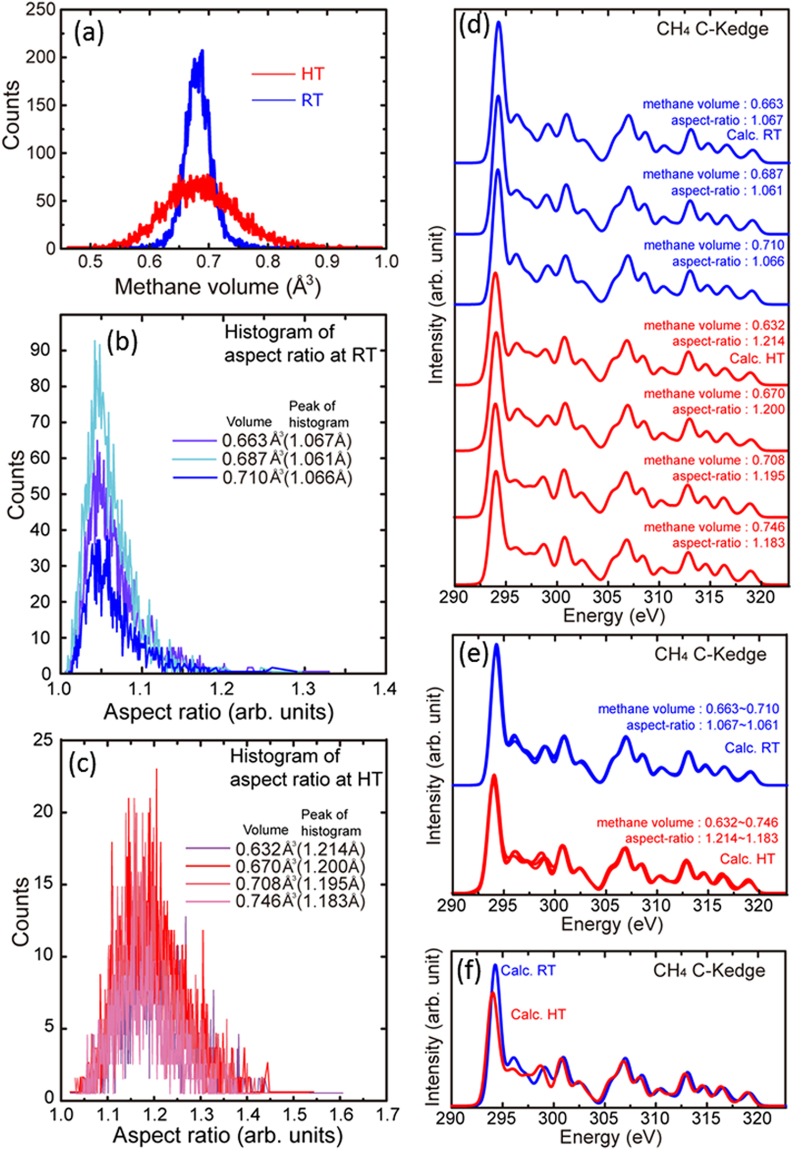
(**a**) Histogram of the CH_4_ volume for the room temperature model (RT: blue line) and high temperature model (HT: red line). Histogram of the aspect ratio of gaseous CH_4_ molecules at (**b**) RT and (**c**) HT. The individual lines correspond to the different volumes, and the averaged aspect ratios. (**d**) Calculated C–K edges of CH_4_ molecules with similar aspect ratios but different volumes at the respective temperatures (RT: 0.663–0.710 Å^3^, HT: 0.632–0.746 Å^3^). (**e**) Overlaid spectra of the C–K edges shown in (**d**) at RT and HT. (**f**) Overlaid spectra of the averaged C–K edges shown in (**d**).

Histograms of the molecular volume and aspect ratio of CH_4_ gas at RT and HT are shown in Fig. 5(a)–(c), and their profiles are broader at HT than at RT. The peak positions of the aspect ratio histograms are 1.06 and 1.20 for RT and HT, respectively, which indicates that the molecular structure of CH_4_ is more distorted at HT than that at RT.

Individual spectra for the RT (blue) and HT (red) models are shown in Fig. 5(d). To separately confirm the effects of volume and the aspect ratio, CH_4_ molecules with a similar aspect ratio (~1.06 for RT and ~1.20 for HT) but different volumes (0.663–0.710 Å^3^ for RT and 0.632–0.746 Å^3^ for HT) were compared. The overlaid spectra of molecules at the same temperature are shown in Fig. 5(e), and confirm that spectra at the same temperature are very similar. This indicates that the spectral profiles are not affected by the change in molecular volume. All of the spectra in Fig. 5(e) are overlaid in Fig. 5(f), and clearly show that the spectral profiles at RT (blue) and HT (red) are different. This difference is similar to that shown in Fig. 4, that is, the first peak becomes less intense and broader, and indicates that the spectral features are more sensitive to changes in the aspect ratio than in the volume.

To summarize, thermal energy was transferred to the O_2_ and CH_4_ gases, and vibration of these molecules was stimulated by the heat transfer. The spectral features of ELNES were sensitive to the strengthened molecular vibration, and vibrational information was successfully extracted from the spectrum using our theoretical calculations.

On the other hand, an open question still remains. That is, the spectral difference between HT and RT in O_2_ was overestimated by the calculation (Fig. 3(a)), whereas that in CH_4_ was well reproduced by the calculation (Fig. 4). To know the origin of this difference, the heat transfer mechanism from the hot filament to the gaseous molecules has to be considered.

Following three phenomena can be considered as the heat transportation mechanism: (a) The gaseous molecules directly contact to the hot filament and the heat is transferred from the filament to the gaseous molecules, and the molecules become “hot” molecules. (b) Multiple collisions of gaseous molecules are taken place near the filament, and the “cold” molecules change to “hot” molecules even though they did not directly contact to the hot filament. This mechanism (b) can heat up only molecules near to the filament. The mechanism (c) is the absorption of the radiant heat from the hot filament. A strong radiant heat is actually emitted from the hot filament (Fig. 1(c)). This mechanism (c) is necessary to heat up the gaseous molecules which are far from the filament.

In general, the mechanisms (a) and (b) are applicable to all kinds of molecules, whereas the mechanism (c) works mainly for polar molecules, such as CH_4_ and CO, because the heat (infrared) is absorbed only by the polar molecule. Namely, the mechanism c) does not work for the non-polar molecules, such as O_2_ and N_2_. In other word, only O_2_ molecules near to the hot filament were heated up by the mechanism b) but they far from the filament did not. Actually, the spectral difference between HT and RT in the calculation is relatively larger than that in the experiment in the case of O_2_ (Fig. 3(a)). On the other hand, the experimental difference was well reproduced by the calculation in the case of CH_4_ (Fig. 4). This difference between O_2_ and CH_4_ is ascribed to the polarity of those molecules. Namely, CH_4_ is polar molecule whereas O_2_ is non-polar molecule, and thus the CH_4_ molecules far from the hot filament were heated up by the absorption of radiant heat.

Based on these consideration, we can conclude that the temperature of CH_4_ is the same as the filament temperature because the experimental spectrum is well reproduced by the calculation (Fig. 4). On the other hand, the spectral difference is overestimated in the calculation in the case of O_2_, indicating that only a part of the O molecule becomes the filament temperature (Fig. 3(a)). This difference would be originating from the heat transportation mechanisms as mentioned above. The heat transportation mechanisms (a) + (b) + (c) work for CH_4_ because it is polar molecule, whereas (c) does not work for non-polar molecule, such as O_2_.

**Figure 6:**
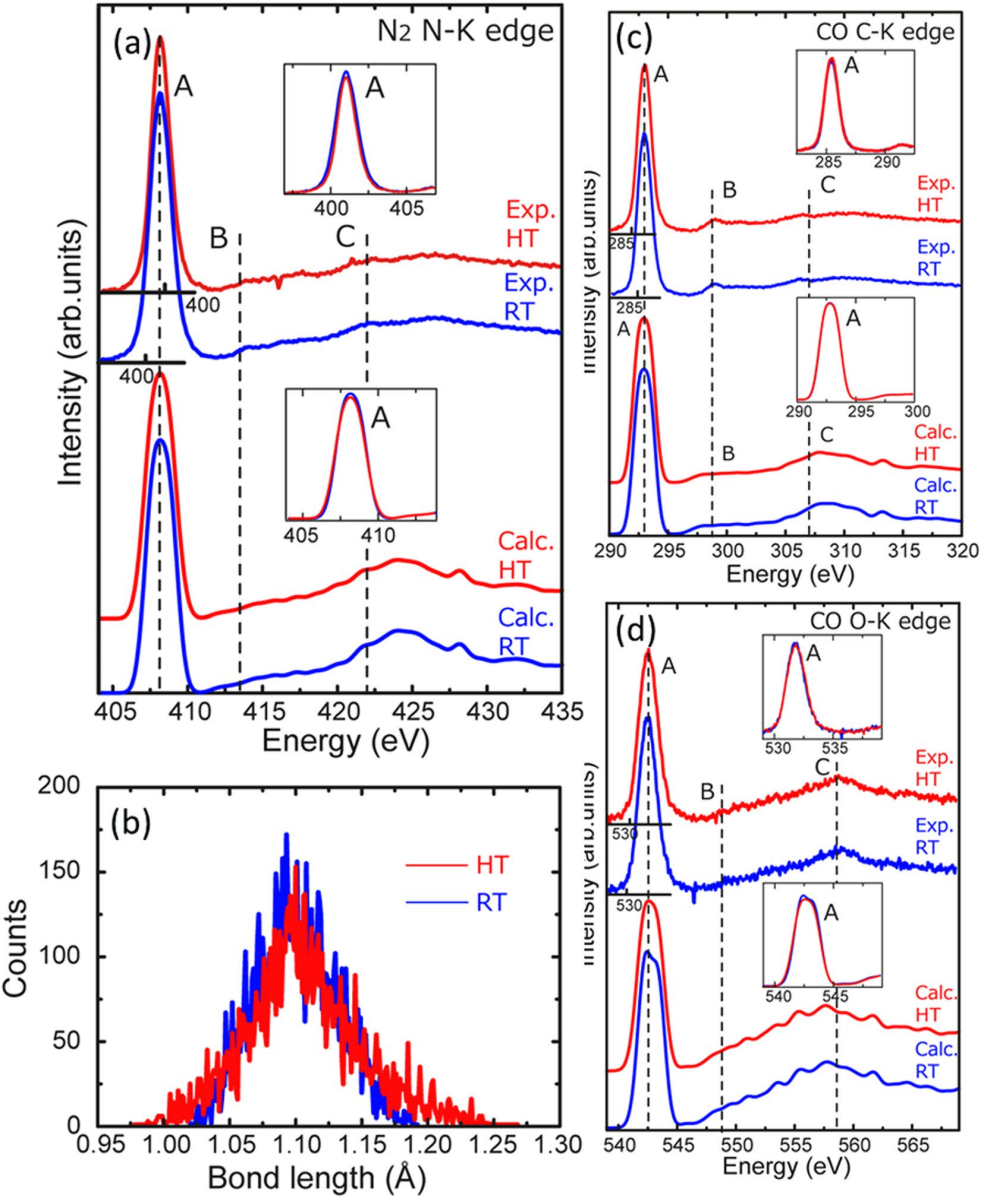
(**a**) Experimental and calculated N–K edges of gaseous N_2_ molecules at room temperature (RT: blue line) and high temperature (HT: red line). (**b**) Histogram of the N≡N bond lengths at RT and HT. Experimental and calculated (**c**) C–K edges and (**d**) O–K edges of gaseous CO molecules at RT and HT.

In contrast to the results for O_2_ and CH_4_, no distinctive changes were observed for N_2_ and CO. The experimental and theoretical N–K edge spectra of N_2_ and the C–K and O–K edge spectra of CO are shown in Fig. 6(a),(c), and (d). Because the electronic structures of N_2_ and CO are similar, all of the spectra have similar profiles; that is, an intense peak A appears at the spectral threshold, and this is followed by small peaks B and C. The experimentally observed features were reproduced in the calculated spectra.

The HT profiles are very similar to those at RT in both the experimental and calculated spectra, as shown in the insets of the figure. Histograms of the N≡N bond length at RT and HT are shown in Fig. 6(b), and unlike those for O_2_ (Fig. 3(b)), the distribution was not affected by the temperature. These results indicate that the dynamic behavior of N_2_ and CO molecules at RT and HT is essentially the same, and the spectral features are thus not sensitive to temperature.

Finally, let us consider the reason why spectral changes were clearly observed for O_2_ and CH_4_ (Figs 3 and 4) but were below the detection limit for N_2_ and CO (Fig. 6). As shown in the histograms, O_2_ and CH_4_ are more sensitive to the temperature change than are N_2_ and CO (Figs 3(b), 5(a)–(c)). The heat transportation mechanism (discussed above) is not important for this sensitivity difference because CO and N_2_ are polar and non-polar molecules as CH_4_ and O_2_, respectively. This can be ascribed to the rigidity of the chemical bonds in the gaseous molecules. The bond energies of N≡N (945 kJ/mol) and C≡O (1071 kJ/mol) are almost double those of O=O (497 kJ/mol) and C–H (416 kJ/mol), which indicates that the molecular structures of N_2_ and CO are rigid and thus insensitive to changes in the kinetic energy of the molecules^21^. In contrast, O=O and C–H are relatively flexible and are thus sensitive to changes in temperature, which means that a larger variety of molecular configurations are generated at increased temperatures.

### Summary

In this study, we have demonstrated the estimation of the dynamic behavior of gaseous molecules using ETEM-ELNES combined with theoretical calculations. Our method successfully identified temperature-induced changes in the molecular vibrations. The relationships between the spectral changes and the dynamic behavior of the gaseous molecules were revealed by combining first-principles calculations and MD simulations. The method presented here has great potential for use in the identification of the dynamic behavior of gaseous molecules with high spatial resolution. Furthermore, our study also suggests that the present method can also be used to measure the local temperature of gaseous molecules.

The sensitivity of our method is affected by the rigidity of the molecule, and our experiment failed to identify the dynamic behavior of the relatively rigid gaseous molecules N_2_ and CO. However, we emphasize that the approach itself is not element- or molecule-dependent, and the sensitivity of the ETEM–ELNES method is rapidly improving with new developments in instrumentation, such as monochromators or detectors^22–25^. We believe that estimation of the dynamic behavior and temperature of many gaseous molecules with improved spatial resolution and sensitivity will be achieved with equipment that will be developed in the near future.

The knowledge obtained in this study will be fundamental for future studies of gaseous reactions using electron microscopy.

## Methods

### Experimental procedure

ETEM-ELNES observations were performed using a cold-field emission environmental transmission electron microscope (HF3300, Hitachi High-Tech) equipped with a gas differential pumping system and a gas-injection heating specimen holder^16^. The accelerating voltage was set to 300 keV, and the energy resolution of ELNES was about 0.7 eV. The measurement point is shown in Fig. 1(a). In this study, we selected four gases: oxygen (O_2_), methane (CH_4_), nitrogen (N_2_), and carbon monoxide (CO), and measured the O–K edge for O_2_, the C–K edge for CH_4_, the N–K edge for N_2_, and the C–K and O–K edges for CO. O_2_ and CH_4_ are industrially essential gases for fuel-cell and hydrogen production^1^, and N_2_ and CO were selected as stable model gases for comparison. The gas pressure in the vicinity of the measurement point was approximately 5–10 Pa. The in-built heater, set to a high temperature (1,273 K), in the transmission electron microscope holder was used to stimulate the molecular vibration of the gases. The temperature of the heater was confirmed using a non-contact radiation thermometer (Fig. 1(b) and (c)). Room temperature (298 K) and high temperature (1,273 K) are represented as RT and HT, respectively.

### Computational procedure

We calculated ELNES spectra using a first-principles planewave basis pseudopotential method based on density functional theory (DFT) with a generalized gradient approximation using CASTEP code^18,19^. The details of the ELNES calculation using CASTEP is described in the previous studies^26,27^. Spin polarization was considered for O_2_. The effect of molecular vibrations was included by constructing gaseous models using an MD simulation. For the MD simulations, 50 Å × 50 Å × 50 Å cubic cells containing 1,000 molecules were first constructed, and simulations using the COMPASSII^28^ force field with forcite code^17^ were performed at RT and HT and 1.014 Pa for 50 ps with a step of 0.01 fs with the NPT ensemble. After convergence of the volume, several snapshots were taken of the simulations and used as the gaseous models.

As an example, Fig. 2(a) shows one of the gaseous models for O_2_ at HT. Although the stable O=O bond length is 1.21 , a large variety of O=O bond lengths were generated by the MD simulation. Figure 2(b) shows a histogram of the O=O bond lengths in the gaseous model. The histogram has a peak at 1.21 Å and the population becomes smaller as the bond length deviates further from the stable length. The dispersion in the O=O bond length reflects the magnitude of the molecular vibrations. We evenly divided the histogram into several sections and extracted representative molecules that had the median bond length of the respective sections. These representative molecules were then separately put into 10 Å × 10 Å × 10 Å supercells and their ELNES spectra were calculated. Then, an averaged ELNES spectrum was constructed by summing the representative spectra with weightings according to the population of each section of the histogram.

This method takes the effects of molecular vibration into consideration, whereas intermolecular interactions are ignored. This assumption is appropriate because the spectral change due to intermolecular interactions can be ignored when a molecule is more than 10 Å away from other molecules^29,30^. We confirmed that the intermolecular distances in our gaseous models are much longer than 10 Å.

In contrast to that used for the diatomic molecules, a different methodology was necessary for the CH_4_ molecule, which has a tetrahedral shape (Fig. 2(c)). To consider the dynamic behavior of CH_4_ molecules, a two-step geometrical consideration was made. In the first step, gaseous models of CH_4_ were constructed from MD simulations using the method described above, and volumes for all CH_4_ molecules were calculated. The volume was defined as that of the tetrahedron made by the four hydrogen atoms in each molecule. Because the volume does not represent the shape of the molecule, we obtained a second distribution for the aspect ratio of the molecules. The aspect ratio was defined as the ratio of the length of the longest H–H edge to that of the shortest H–H edge of a CH_4_ molecule. The aspect ratio is 1 when the molecule is a regular tetrahedron, and becomes more than 1 when it is distorted. To obtain the aspect ratio distribution, the histogram of the volume was divided into several regions, and the distributions of the aspect ratios of the molecules in the respective regions were calculated. Figure 2(d) shows a schematic representation of the two-step analysis.

After the two types of histograms were obtained, a single representative molecule was selected from each region in the aspect ratio histogram. These molecules were put into a 10 Å × 10 Å × 10 Å supercell, and the C–K edge of the representative molecule from each region was calculated. Then, the averaged ELNES spectrum was calculated using the ratios obtained from the populations in the volume histogram.
